# Pediatric Oncology in Nigeria: A Panoramic View

**DOI:** 10.1200/JGO.18.00231

**Published:** 2019-07-03

**Authors:** Adeseye Michael Akinsete, Babatunde Adeniran Odugbemi, Gbemisola Eniola Ogundowole, Uchechukwu Udochukwu Anene-Nzelu, Edamisan Temiye, Adebola Akinsulie

**Affiliations:** ^1^ University of Lagos College of Medicine, Lagos, Nigeria; ^2^ Lagos State University College of Medicine, Lagos, Nigeria; ^3^ Lagos University Teaching Hospital, Lagos, Nigeria

## Abstract

**PURPOSE:**

A large number of children still die as a result of cancer in low- to middle-income countries, and factors such has poor infrastructure, inadequate human resources, and poorly developed health insurance are responsible for most of these deaths. Nigeria is a country with a young population and a struggling health system. We aimed to survey pediatric oncologists in Nigeria using an online survey instrument.

**METHODS:**

We surveyed the national group of pediatric oncologists using an instrument designed to assess manpower availability, infrastructural support, support services, and presence of radiotherapy and medications.

**RESULTS:**

A total of 14 institutions responded, represented by 24 oncologists of the 42 oncologists on the platform, with a response rate of 57.1%. Most of the oncologists had practiced for more than 10 years, but only two institutions had a dedicated pediatric oncology ward. There was no population-based pediatric oncology tumor registry. Molecular diagnostic capability was not available, nor was a structurally efficient radiotherapy support service. The centers also struggled with inadequate blood and blood product provision.

**CONCLUSION:**

Pediatric oncology services in Nigeria are still grappling with weak human capital, poorly developed infrastructure, weak regional and national referral systems, and poor support services.

## INTRODUCTION

An estimated 200,000 children are diagnosed annually with cancer in low- to middle-income countries (LMICs).^1^ Although survival rates in most cancers in the developed world are greater than 80%, in LMICs, the survival rates is approximately 20%.^2,3^ Pediatric oncology care in resource-constrained settings is restricted by many challenges, including poor infrastructure, inadequate and poorly trained manpower, absence of chemotherapeutic agents, inadequate or inappropriate diagnostic support, and poorly developed health insurance.^2,4^ It is estimated that children make up 41% of the African population, and the prevalence of cancer may continue to increase as a result of a more robust and functional primary health care framework and associated rural urban drift.^5^ There is only one functional population-based pediatric cancer registry in Africa, with most registries operating out of tertiary hospitals.^6^ This creates a huge problem in planning and providing care, because data are unreliable in most resource-constrained settings. Data from the registry show that leukemia and lymphoma were the commonest malignancies observed, and they affected more males than females.^7^

**CONTEXT**

**Key Objective**
Pediatric oncology in Nigeria and most low- to middl- income countries has high morbidity and mortality statistics. There is a dearth of human capital as well as physical infrastructure. The study objective was to document what is currently available in the pediatric oncology landscape in Nigeria.
**Knowledge Generated**
The study showed the inequitable distribution of services in the country, the silo mentality of practitioners, and the poor infrastructural development in the sector.
**Relevance**
This study can assist with development of an efficient referral system, which will improve time to diagnosis and treatment and also strengthen the pediatric oncology ecosystem.

Cancer care is a complex collaborative mix involving several specialties. Manpower, infrastructure, technology, strong multidisciplinary interaction, and parent support groups within a solely dedicated pediatric institution must be integrated to achieve great results. Nigeria is a nation with an estimated population of 180 million and a median age of 17.9 years, with approximately 51.4% living in urban areas.^8^ Currently in Nigeria, there is no population-based pediatric cancer registry and no national database or surveillance mechanism for pediatric cancer. Furthermore, there are not many standalone pediatric institutions or pediatric oncology centers in the country. The emphasis of government has been on preventive and curative services, mainly for types of cancer occurring in adults, such as cervical, breast, prostate, colon, and lung cancers. In 2011, pediatric oncologists came together under one umbrella, the Nigerian Society of Pediatric Oncology (NISPO), to chart a focused path and improve the practice of pediatric oncology in the country. Membership in the society is voluntary, and members are expected to pay an annual membership fee to run the society. The society meets annually to discuss and review protocols and practice guidelines. This study presents an overview of the resources currently available for pediatric oncology in Nigeria.

## METHODS

This was a cross-sectional survey of pediatric oncologists who were in the NISPO database. This survey was conducted from April 2018 to September 2018. Different institutions in the group were identified, and a structured pretested questionnaire was sent online to oncologists in the group. The questionnaire had six sections, comprising demographics, human capital, physical infrastructure, and presence of radiotherapy, medications, and support services (Data Supplement).

There were 42 members of the society registered at the time of the survey. Respondents completed and submitted the survey online. Exemption was obtained from the Health Research and Ethics Committee of the Lagos University Teaching Hospital. Permission was obtained from the secretariat of NISPO to use its roll of member names for the research. After retrieving the responses, multiple questionnaires from same institution were reviewed, and follow-on telephone interviews were conducted to clarify inconsistent information from oncologists in the same institution.

Statistical analysis was performed with the aid of statistical software for the social sciences (SPSS version 20; SPSS, Chicago, IL). Frequency tables were generated for the responses.

## RESULTS

A total of 14 questionnaires were used for the analysis, representing 14 institutions. Multiple responses from the same institution were harmonized to prevent duplication of information. A total of 24 oncologists of the 42 oncologists on the roll responded, corresponding to a 57.1% response rate. Five responses were voided because of incomplete information; the five respondents excluded from further analysis only completed the biodata information section. All oncologists who responded were included in the survey regardless of years of practice. Regarding the voided questionnaires, three of the respondents were from southwestern Nigeria, one was from southern Nigeria, and one was from northwestern Nigeria. Most oncologists had practiced for longer than 10 years, and they worked in tertiary institutions across the country. Only one oncologist practiced in the private sector.

**FIG 1:**
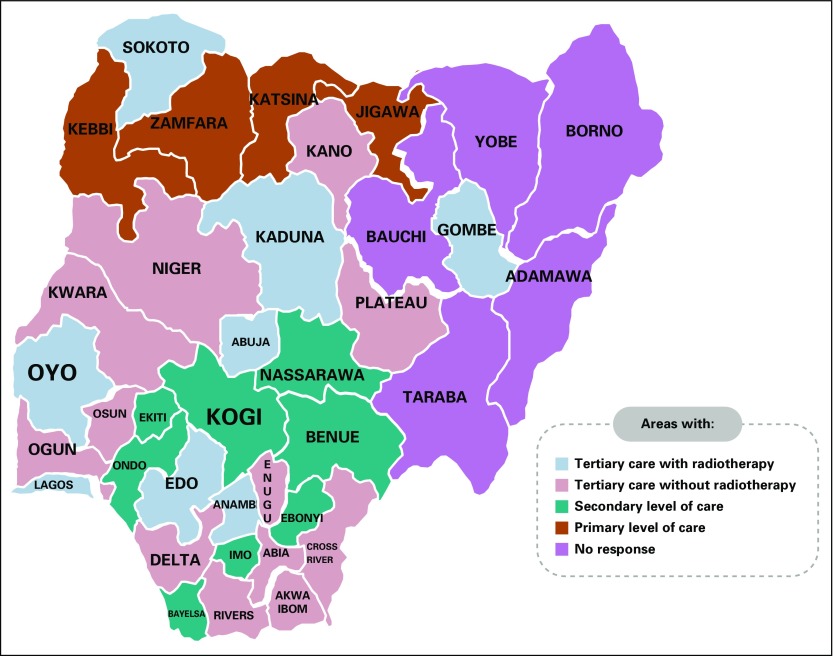
Distribution of pediatric oncology services in Nigeria.

Only 50% of the institutions surveyed had some form of multidisciplinary team (MDT) meeting. None of the institutions had a population-based pediatric oncology tumor registry. Approximately half had hospital-based tumor registries that were not pediatric based. The same number had documented protocols, and all those who used documented protocols reviewed them periodically. The protocols used were adapted from the International Society of Pediatric Oncology, Children’s Oncology Group, Berlin-Frankfurt-Münster, and the United Kingdom. The protocols used were largely pediatric. Efforts are ongoing to adopt national protocols for uniformity of practice. Results are listed in Tables 1 to 5 and Figure 1.

**TABLE 1:**
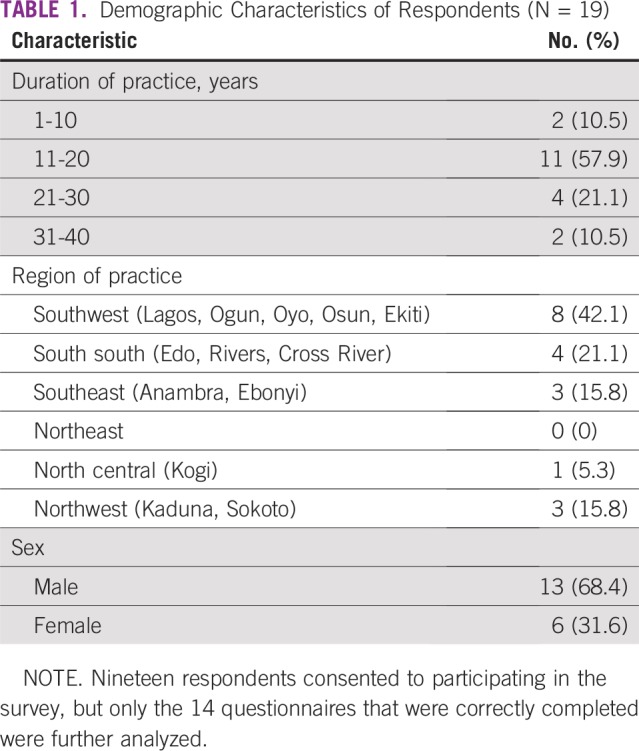
Demographic Characteristics of Respondents (N = 19)

**TABLE 2:**
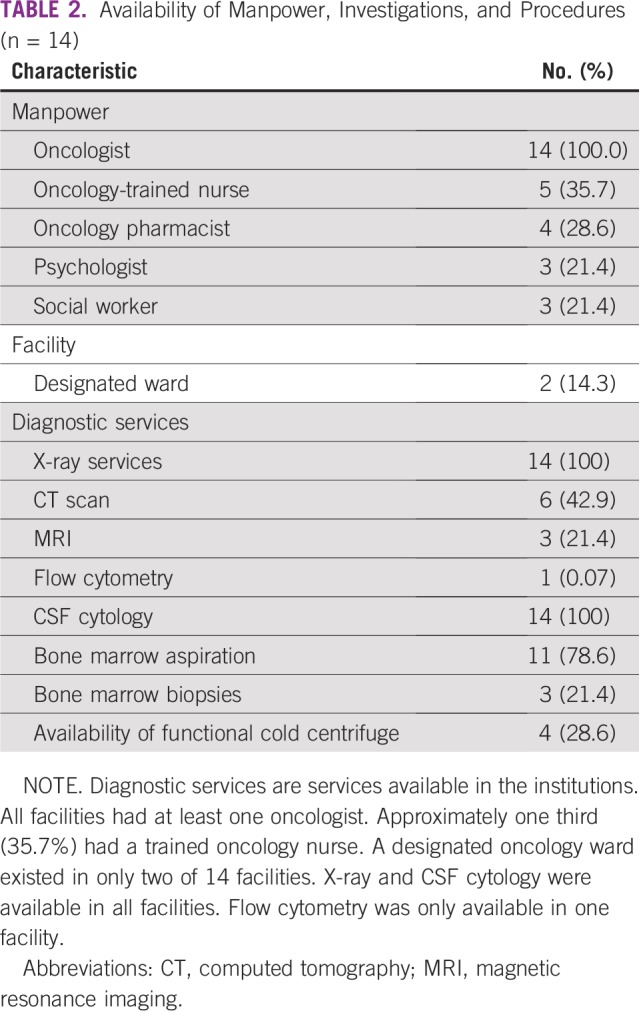
Availability of Manpower, Investigations, and Procedures (n = 14)

**TABLE 3:**
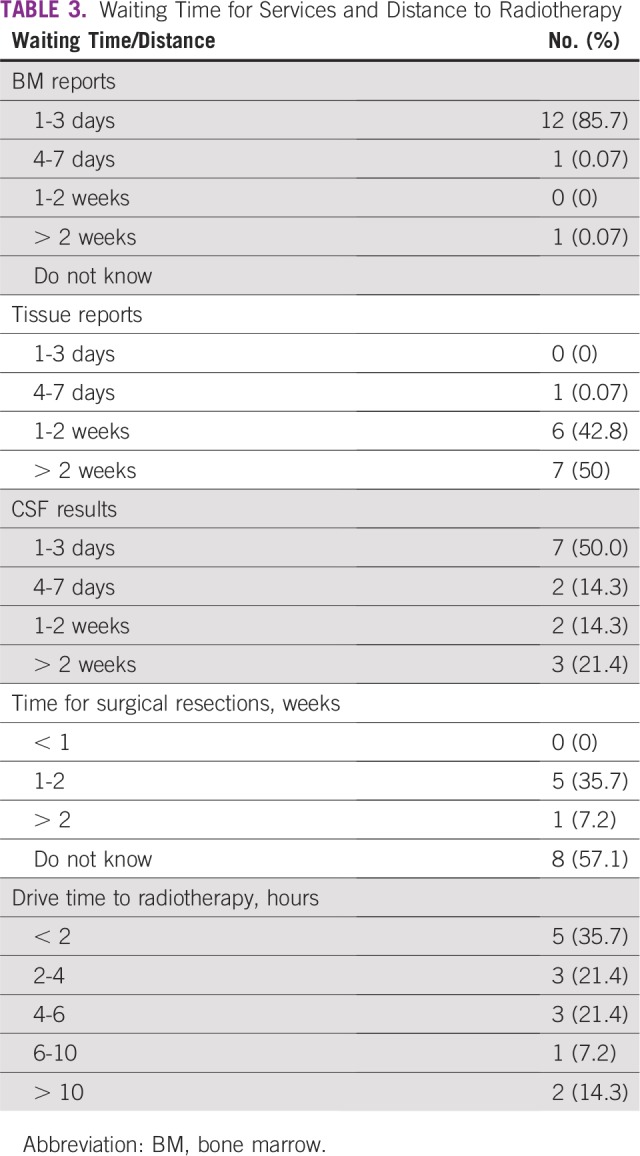
Waiting Time for Services and Distance to Radiotherapy

**TABLE 4:**
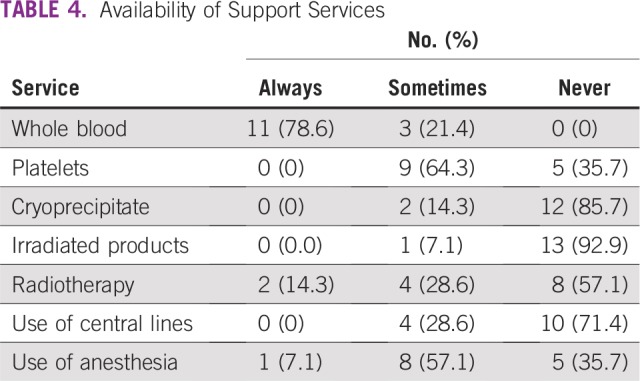
Availability of Support Services

**TABLE 5:**
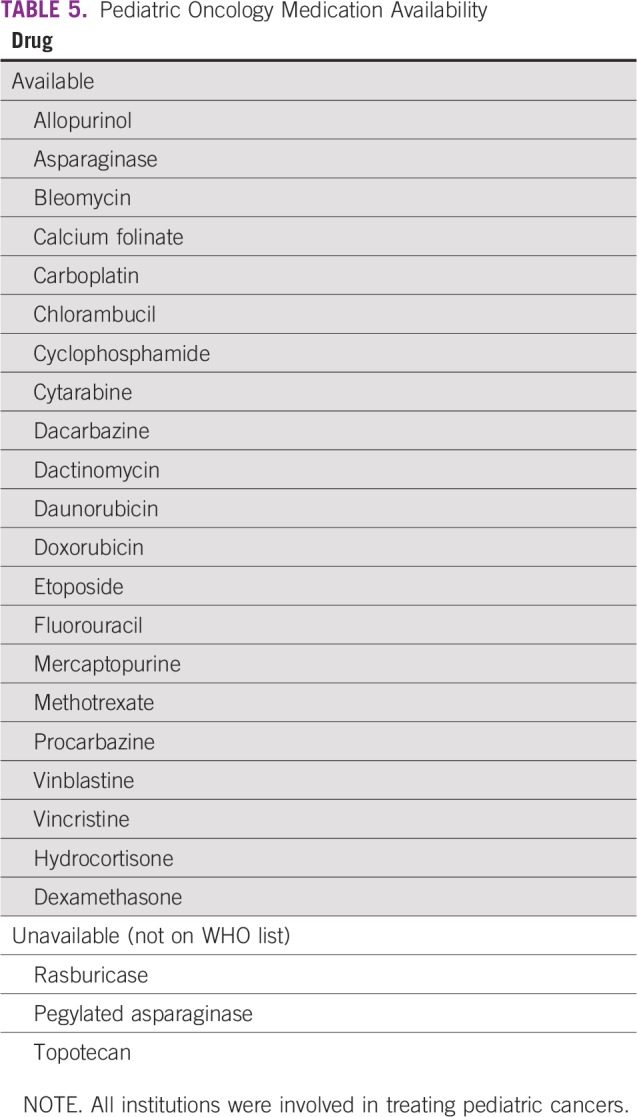
Pediatric Oncology Medication Availability

## DISCUSSION

An attempt at identifying the availability of human and infrastructural resources is a right step in the improvement of pediatric cancer services in Nigeria. Nigeria grapples with poor infant and child mortality statistics, and there has not been much attention paid to the contribution of noncommunicable diseases to these figures.^9^

Our survey revealed the inequitable distribution of human resources in the nation. Nigeria is divided into six geopolitical zones across the north and south of the country. Most of the oncologists surveyed were experienced, having practiced for more than 10 years, but they were unequally distributed across the nation. It was also discovered that no oncologist responded from the northeastern part of the country. This part of the country has been grappling with problems of insurgency for the last 6 years. The fact that only a meager 10% of the respondents had practiced for between 5 and 10 years may be a sign of a lack of interest among trainees in pediatric oncology, indicating an emerging problem. MDT care has been associated with improved quality of care, defined care pathways, and improved treatment outcomes, even among patients with advanced disease.^10^ Unfortunately, a majority of the institutions surveyed did not have dedicated trained pediatric oncology nurses. This will affect the quality of care delivered to children and ultimately affect outcomes. Other professionals, such as oncology pharmacists, psychologists, nutritionists, and social workers, were scarcely available across the pediatric oncology landscape. None of the hospitals had dedicated professionals for children with cancer.

All respondents worked in hospitals that had more than 200 pediatric beds, but only two of the institutions had designated pediatric oncology wards. Interestingly, all the institutions had designated adult oncology wards. A designated pediatric oncology ward has been described as a necessary component in the delivery of quality care to children and their parents.^6^ The lack of a designated ward implies an inability to deliver highly focused and unique care to this group of children. Diagnosis forms the bedrock of modern oncology practice. All the institutions could perform basic radiologic services; however, fewer than half of the institutions had access to computed tomography scan facilities. This will lead to prolonged time to diagnosis, increased anxiety among the children and their parents, and poor quality of care. Fewer than a quarter of the institutions surveyed had access to magnetic resonant imaging. It was observed that a large swath of land covering more than 276,959 km^2^ and spanning several states was without any form of advanced imaging services, any pediatric oncologists, and any form of structured care. This is the orange area highlighted on the map of Nigeria (Fig 1).

In a nation of approximately 197 million people covering 923,768 km^2^, there is only one flow cytometer for the classification of hematologic malignancies. Flow cytometry is an important adjunct in the diagnosis and monitoring of those with hematologic malignancies.^11^ This situation will translate to imprecision with regard to the diagnosis of leukemia. Relying on only microscopic diagnosis of bone marrow smears is fraught with error and imprecision. Furthermore, monitoring minimal residual disease at a microscopic level is not possible without the aid of a flow cytometer. Clinicians are thus at a disadvantage, and patients are obviously not getting the best from the system. Molecular diagnostic investigations are carried out outside the country, usually in South Africa or India. Such investigations are expensive and out of reach for most patients; when they are available, it is usually between 7 to 10 days before results are received by clinicians. All those who access this service have to pay out of pocket. Traditional bone marrow aspirations are performed by most of the institutions surveyed, but few perform trephine biopsies. Trephine biopsies require a bit more skill and technique, and this may be the reason for its low uptake among the surveyed clinicians.

Multidisciplinary care has been linked to improved survival, even in advanced disease.^10^ Timely receipt of reports, prompt provision of services, and smooth interaction among complex teams is one of the highlights of good pediatric oncology services. Generally, across the country, timely reports are received for bone marrow aspirates; however, this is not the same for tissue reports. It takes on average 7 to 14 days for reports to be retrieved for tissue samples. This introduces the potential for progressive disease at the time of diagnosis. Immunohistochemistry is not routinely performed for all tissues processed across the country. The general trend is for the parents or caregivers to pay separately for immunohistochemistry. Because most parents are paying out of pocket, they routinely settle for simple histologic diagnosis, which is limited in scope and has poor specificity and sensitivity. Surgical resections were also not promptly performed in all the institutions surveyed. A small fraction of the institutions surveyed reported that surgical resections occurred within 2 weeks; most were not sure when resections were going to occur. The time lag between diagnosis and surgical resection in Nigeria is a result of most institutions administering neoadjuvant chemotherapy before surgery despite clinical stage. This occurs because of a peculiarity of the patients seen in most of the institutions. A good number of patients abandon treatment after surgery, so a course of chemotherapy administered earlier provides some coverage. For the institutions where surgeries were not performed within defined timeframes, clinicians reported that surgeries were often not definite because of problems ranging from patient factors to operational issues to infrastructural needs. The problems of disorganized care revealed in this survey largely result from a lack of MDT meetings. Half of the respondents said they had MDT meetings, but they were not regular. MDT meetings have been reported to improve patient care and outcomes.^12,13^ They represent the communication room and strategic driver of oncology care.^13^ MDT meetings did not occur in any of the units that complained of long intervals between diagnosis and surgical resection. This was also replicated in the time reports received from the laboratories. Therefore, it can be safely assumed that MDT meeting introduction will improve time to diagnosis and intervention, barring unforeseen circumstances.

The management of children with cancer requires the use of blood and blood products, as a result of either the malignancy or subsequent therapy. It is still a challenge to get the required quantities of blood for patients in need. Reasons include lack of blood donors, unwillingness of family members to donate blood, request for financial inducements for blood donation, lack of funds, and inadequate consumables.^14^ In this setting, obtaining the required quantities of blood and platelets is a major problem. Fewer than 10 cold centrifuges are functional in the southern part of the country, catering to a population of more than 80 million, with fewer still upcountry, catering to a much larger population. The implication is the loss of life as a result of thrombocytopenia, the rate of which is particularly high among children with hematologic malignancies, especially acute myeloid leukemia, in Nigeria.^15^ Stem-cell transplantation is only performed at one institution in Nigeria, and therefore, the uptake of irradiated blood and blood products was expectedly low. Central venous catheters are the standard in the developed world, but this is not a common practice in Nigeria. Most of the institutions still used peripheral access, which increases the pain and discomfort the children must endure. Considering that these children have to stay long periods in the hospital, frequent changes of peripheral cannulas and thrombophlebitis are not uncommon. Chemotherapeutic port devices are not used at all in any of the institutions. Combination of chemotherapy and radiotherapy is useful in the care of children with oncology. Many solid tumors require both forms of therapy. At the time of the survey, there were six linear accelerators (LINACs) installed in Nigeria. Unfortunately, only two were functional at the time of the survey. Therefore, all other institutions had to fashion ways of moving the children in need of radiotherapy to these institutions. The relative distances were a challenge for most parents, and this further worsened the outcomes. The LINACs break down often because of heavy patient load, and children bear the brunt of this malfunction. There was an institution with a cobalt 60 machine, which served a population of close to 35 million people. Expectedly, children are sent to the institutions with LINACs for therapy. This means several hours to days of therapy as well as more funds expended on accommodation. All patients who access radiotherapy care do so out of pocket.

Regarding medication availability, all of the drugs on the WHO essential drugs list are available in the country. The challenge of most caregivers is the cost of some of these medications, because patients must pay out of pocket. However, the newer immunotherapeutic agents are not commonly available locally. This has led to protocol modifications generally among several of the institutions surveyed. We found that drugs were generally more available and accessible in the southern parts of the country when compared with the north. All the units upcountry usually refer patients to Lagos, the commercial nerve center of Nigeria, whenever there is a need for immunotherapy.

Pediatric oncology care will benefit from a more cohesive and focused approach. A national pediatric oncology clinical care commission will provide benefit for the specialty and enhance practice, access to care, and survival. We recommend the following: creation of a national pediatric oncology tumor registry as a first step in data gathering and improving outcomes, deliberate action to address the issues of human capital development and physical infrastructure, establishment of a national pediatric oncology referral system along regional lines, and encouragement of MDT meetings as a minimum monthly requirement in all institutions, along with regional MDTs.
